# Supplementary material for: Managing Difficulties of Microsatellite Instability Testing in Endometrial Cancer-Limitations and Advantages of Four Different PCR-Based Approaches
Source: Cancers (Basel). 2021 Mar 12;13(6):1268. doi: 10.3390/cancers13061268 (PMC8000432; doi:10.3390/cancers13061268)
Supplement: Supplementary file 1 [file cancers-13-01268-s001.pdf]

cancers

MDPI

Supplementary Materials: Managing Difficulties of Microsatellite Instability Testing in Endometrial Cancer-Limitations and Advantages of Four Different PCR-Based Approaches

Janna Siemanowski [1,*,#], Birgid Schömig-Markiefka [1,#], Theresa Buhl [1], Anja Haak [2], Udo Siebolts [2], Wolfgang Dietmaier [3], Norbert Arens [4], Nina Pauly [5], Beyhan Ataseven [5,6], Reinhard Büttner [1] and Sabine Merkelbach-Bruse [1]

Table 1. Clinical-demographical characteristics of all samples.

Sample	Cohort	Age	Sex	Diagnosis	MSI-status
1	1	68	f	EEC	MSI-H
2	1	74	f	EEC	MSI-H
3	1	56	f	EEC	MSI-H
4	1	56	f	EEC	MSI-H
5	1	74	f	EEC	MSI-H
6	1	64	f	EEC	MSI-H
7	1	45	f	EEC	MSI-H
8	1	85	f	EEC	MSI-H
9	1	62	f	EEC	MSI-H
10	1	84	f	EEC	MSI-H
11	1	47	f	EEC	MSI-H
12	1	87	f	EEC	MSI-H
13	1	77	f	EEC	MSI-H
14	1	65	f	EEC	MSI-H
15	1	57	f	EC	MSI-H
16	1	68	f	EC	MSI-H
17	1	55	f	EEC	MSI-H
18	1	59	f	EEC	MSI-L
19	1	60	f	EEC	MSS
20	1	71	f	EEC	MSS
21	1	57	f	clearcell EC	MSS
22	1	70	f	EEC	MSS
23	1	55	f	EEC	MSS
24	1	55	f	EEC	MSS
25	1	70	f	EEC	MSS
1	2	55	f	EEC	MSI-H
2	2	58	f	EEC	MSI-H
3	2	63	f	EEC	MSI-H
4	2	n.a.	f	EEC	MSI-H
5	2	n.a.	f	EEC	MSI-H
6	2	n.a.	f	EEC	MSI-H
7	2	65	f	EEC	MSI-H
8	2	n.a.	f	EEC	MSI-H
9	2	n.a.	f	EEC	MSI-H

10	2	n.a.	f	EEC	MSI-H
11	2	n.a.	f	EEC	MSI-H
12	2	n.a.	f	EEC	MSI-H
13	2	n.a.	f	EEC	MSI-H
14	2	n.a.	f	EEC	MSI-H
15	2	56	f	EEC	MSI-H
16	2	n.a.	f	EEC	MSI-H
17	2	n.a.	f	EEC	MSI-H
18	2	63	f	EEC	MSI-H
19	2	n.a.	f	EEC	MSI-H
20	2	n.a.	f	EEC	MSI-H
21	2	n.a.	f	EEC	MSI-H
22	2	n.a.	f	EEC	MSI-H
23	2	76	f	EEC	MSS
24	2	42	f	EEC	MSI-H
25	2	63	f	EEC	MSI-H
26	2	58	f	EEC	MSI-H
27	2	n.a.	f	EEC	MSI-H
28	2	49	f	EEC	MSI-H
29	2	56	f	EEC	MSI-H
30	2	60	f	Ov EC	MSI-H
31	2	40	f	EEC	MSI-H
32	2	71	f	EEC	MSI-H
33	2	55	f	EEC	MSI-H
34	2	60	f	EEC	MSI-H
35	2	53	f	EEC	MSI-H
36	2	78	f	EEC	MSI-H
37	2	33	f	EEC	MSI-H
38	2	66	f	EEC	MSI-H
39	2	55	f	EEC	MSI-H
40	2	60	f	EEC	MSI-H
41	2	83	f	EEC	MSI-H
42	2	80	f	EEC M	MSI-H
43	2	59	f	EEC	MSI-H
44	2	88	f	EEC	MSI-H
45	2	n.a.	f	EEC	MSI-H
46	2	71	f	EEC M	MSI-H
47	2	n.a.	f	EEC	MSI-H
48	2	71	f	EEC	MSI-H
49	2	45	f	EEC	MSI-H
50	2	70	f	EEC	MSI-H
51	2	38	f	EEC	MSI-H
52	2	60	f	EEC	MSI-H
53	2	n.a.	f	EEC	MSS
54	2	58	f	EEC	MSS
55	2	65	f	EEC	MSS

56	2	61	f	EEC	MSS
57	2	62	f	EEC	MSS
58	2	n.a.	f	EEC	MSS
59	2	66	f	EEC	MSS
60	2	71	f	EEC	MSS
61	2	69	f	EEC	MSS
62	2	59	f	EEC	MSS
63	2	83	f	EEC	MSS
64	2	67	f	EEC	MSS
65	2	73	f	EEC	MSS
66	2	58	f	EEC	MSS
67	2	43	f	EEC	MSS
68	2	69	f	EEC	MSS
69	2	58	f	EEC	MSS
70	2	79	f	EEC	MSS
71	2	45	f	EEC	MSS
72	2	84	f	EEC	MSS
73	2	56	f	EEC	MSS
74	2	76	f	EEC	MSS
75	2	56	f	EEC	MSS
1	none	57	f	CRA Rectum	MSI-H
2	none	57	f	CRA Rectum	MSI-H
3	none	75	f	CRA M	MSI-H
4	none	51	m	CRA Coecum	MSI-H
5	none	59	m	CRA -	MSI-H
6	none	70	m	CRA Coecum	MSI-H
7	none	78	f	CRA M	MSI-H
8	none	58	f	CRA C asc	MSI-H
9	none	72	f	CRA C asc	MSI-H
10	none	87	f	CRA C asc	MSI-H
11	none	97	f	CRA -	MSI-H
12	none	77	f	CRA Coecum	MSI-H
13	none	82	f	CRA C trans	MSI-H
14	none	41	m	CRA Coecum	MSI-H
15	none	94	f	CRA C asc	MSI-H
16	none	76	m	CRA -	MSI-H
17	none	90	f	CRA C asc	MSI-H
18	none	73	f	CRA -	MSI-H
19	none	64	f	CRA C asc	MSI-H
20	none	88	m	CRA C asc	MSI-H
21	none	62	m	CRA -	MSI-H

[s1]**(f)** Female; **(m)** male; **(n.a.)** no data available; **(EEC)** endometrioid endometrial carcinoma; **(EC)** endometrioid adenocarcinoma; **(EEC M)** endometrioid endometrial carcinoma metastasis **(Ov EC)** ovarian endometrioid adenocarcinoma; **(CRA)** colorectal adenocarcinoma; (CRA -) colorectal adenocarcinoma without localization; **(CRA M)** colorectal adenocarcinoma metastasis; **(CRA C asc)** colorectal adenocarcinoma - ascending colon; **(CRA C trans)** colorectal adenocarcinoma - colon transversum.
